# Resveratrol Impedes the Stemness, Epithelial-Mesenchymal Transition, and Metabolic Reprogramming of Cancer Stem Cells in Nasopharyngeal Carcinoma through p53 Activation

**DOI:** 10.1155/2013/590393

**Published:** 2013-04-29

**Authors:** Yao-An Shen, Chien-Hung Lin, Wei-Hsin Chi, Chia-Yu Wang, Yi-Tao Hsieh, Yau-Huei Wei, Yann-Jang Chen

**Affiliations:** ^1^Institute of Biochemistry and Molecular Biology, National Yang-Ming University, Taipei 112, Taiwan; ^2^Institute of Clinical Medicine, National Yang-Ming University, Taipei 112, Taiwan; ^3^Department of Life Sciences and Institute of Genome Sciences, National Yang-Ming University, Taipei 112, Taiwan; ^4^Department of Medicine, Mackay Medical College, New Taipei City 252, Taiwan; ^5^Department of Pediatrics, Taipei City Hospital, Renai Branch, Taipei 106, Taiwan

## Abstract

Cancer stem cells (CSCs) are able to self-renew and are refractory to cancer treatment. To investigate the effects of resveratrol on CSCs of nasopharyngeal carcinoma (NPC), we employed a behavior selection strategy to isolate CSCs based on radioresistance, chemoresistance, and tumor sphere formation ability. These NPC CSCs displayed stem cell properties and underwent metabolic shift to predominately rely on glycolysis for energy supply. Intriguingly, we found that resveratrol turned off the metabolic switch, increased the reactive oxygen species (ROS) level, and depolarized mitochondrial membranes. These alterations in metabolism occurred concomitantly with the suppression of CSC properties including resistance to therapy, self-renewal capacity, tumor initiation capacity, and metastatic potential in NPC CSCs. We found that resveratrol impeded CSC properties through the activation of p53 and this effect could be reversed by knockdown of p53. Furthermore, resveratrol suppressed the stemness and EMT through reactivating p53 and inducing miR-145 and miR-200c, which were downregulated in NPC CSCs. In conclusion, we demonstrated that resveratrol employed the p53 pathway in regulating stemness, EMT, and metabolic reprogramming. Further investigation of the molecular mechanism of p53 activation by resveratrol may provide useful information for the development of novel therapies for cancer treatment through targeting to CSCs.

## 1. Introduction

Nasopharyngeal carcinoma (NPC) is a distinctive type of head and neck cancer with high prevalence rates in Southeast China and Taiwan. Unlike other head and neck cancers, most cases of NPC have high tendency to invade surrounding tissues and metastasize to regional lymph nodes at an early stage. Moreover, most mortality of NPC patients is due to local recurrence and distant metastases [1]. Although chemotherapy and radiotherapy can improve survival rate, the prognosis remains poor for patients with relapse or metastatic diseases [2]. Therefore, in order to effectively identify the target of therapy, the underlying mechanisms that lead to NPC recurrence and metastasis must be clarified.

Emerging evidence substantiated the notion that cancer stem cells (CSCs) or tumor-initiating cells are able to self-renew and regenerate tumor mass. CSCs are refractory to therapy by dint of their quiescent characteristics and expressing ATP-binding cassette transporters [3]. In addition, epithelial-mesenchymal transition (EMT) imparts not only metastasis capacity but also the CSC properties to tumor cells [4, 5]. Thereby, the discovery of potential drugs targeting at CSCs may solve the clinical curative difficulties such as therapeutic resistance, recurrence, and metastasis.

Accumulated evidence has shown that phenolic compounds, such as curcumin, epigallocathechin gallate, and resveratrol, may have potential inhibitory effects on CSCs and prevent tumor invasion and metastasis [6–9]. Among natural polyphenols, resveratrol (3,5,4′-trihydroxy-trans-stilbene) is present mostly in red wine that has pharmacological properties including antiaging, anti-inflammation, and antitumor capacity. Recent studies revealed that resveratrol induced NPC cells apoptosis through activating multiple apoptotic pathways [10]. Furthermore, resveratrol also inhibited CSC properties in pancreatic cancer, breast cancer, and glioblastoma [11–13]. Resveratrol could efficiently suppress the invasion and metastasis of tumor cells through reversing the EMT process in lung and breast cancers [14, 15]. It also reduced the self-renewal capacity and stemness gene signatures of CSCs in head and neck cancers [9].

However, the mechanisms pertaining to resveratrol mediated signaling pathways in CSCs remained unclear. In this study, we aimed to use NPC CSCs as a model to dissect the regulation of resveratrol in stemness, EMT, and metabolic signatures of CSCs and to explore the potential therapeutic targets in NPC CSCs.

## 2. Material and Methods

### 2.1. Cell Culture

Three human NPC cell lines, TW01, TW06, and HONE-1, were used in this study [16, 17]. All NPC cell lines were cultured in complete Dulbecco's Modified Eagle Medium (DMEM), supplemented with 1% sodium pyruvate, 1% nonessential amino acids, 1% of penicillin-streptomycin, and 10% fetal bovine serum (FBS) (Invitrogen, Carlsbad, CA) at 37°C with humidified 5% CO_2_.

### 2.2. CSC Behavior Selection

To acquire high ratio of CSCs from NPC cell lines, we developed a behavior selection method according to CSC's characteristics. The parental cells would be selected through irradiation treatment, sphere formation and side population serially. Firstly, irradiation selection was performed as described previously [18]. Radioresistant clones were established after four rounds of 11 Gy irradiation at 37.9 mGy/s using Rad Source RS 2000 X-ray biological irradiator (Rad Source Technologies, Inc., Suwanee, GA). The radioresistant capacity of these cells was measured by clonogenic assay. Total of 5 × 10^3^ cells were seeded and treated with 10 Gy irradiation. After 10 days, colonies were fixed with 3 : 1 ratio of methanol and glacial acetic acid, stained with 2% crystal violet, and counted under a phase-contrast microscope. After the radioresistant phenotype was corroborated, the selected radioresistant cells were then proceed to tumor sphere selection. Tumor sphere selection was performed as described previously [19]. Cells were seeded into 0.4% soft agar coated petri dish with serum-free DMEM. The soft surface rendered the cells unable to attach and formed tumor spheres after 10 days. Finally, CSCs were isolated with side population selection by using cells from tumor spheres. Side population assay was performed as described previously [20]. Briefly, cell concentration was adjusted to 10^6^ cells/mL with DMEM medium supplemented with 2% FBS and then treated with Hoechst 33342 (5 *μ*M, Sigma-Aldrich) and incubated in a 37°C incubator for 90 min. After twice wash with PBS, propidium iodide (PI) (2 *μ*g/mL, Sigma-Aldrich) was added to exclude dead cells. Cells were kept at 4°C in the dark before sorting by BD FACSAria flow cytometer (BD Biosciences, San Jose, CA). The side population gating requires control experiments with the ATP-binding cassette (ABC) transporter inhibitor. As a control, cells were incubated with fumitremorgin C (FTC, 10 *μ*M, Sigma-Aldrich) for 30 min at 37°C prior to and during Hoechst dye staining. FTC would block the ABC transporters from extruding Hoechst dye. The side population cells would exhibit high fluorescence when treated with FTC to block the efflux of Hoechst dye. We then compared the pattern with or without the treatment of FTC to define and isolate side population cells.

### 2.3. Survival Fraction Analysis

Survival fractions were performed by comparing the number of survival colonies of the control with the test samples under resveratrol treatment (10, 25, and 50 *μ*M, Sigma-Aldrich) and irradiation treatment (2, 4, 6, 8, 10, and 12 Gy), respectively. Surviving fraction was calculated using the number of colonies divided by the number of cells seeded and corrected with the plating efficiency. Plating efficiency was calculated by dividing the number of colonies with the number of cancer cells seeded.

### 2.4. Soft Agar Assay

The plates were decoated with 1.2% soft agar as a base. After the agar solidified, 1 × 10^4^ cells with or without 50 *μ*M resveratrol treatment were mixed with 0.4% soft agar and seeded on the base. The colonies grown in soft agar with diameters >500 *μ*m were counted after two weeks.

### 2.5. Wound Healing Migration Assay

We utilized ibidi culture inserts (ibidi GmbH, Munich, Germany) for wound healing migration assay and performed according to the manufacture's protocol. The insert contains two reservoirs separated by a 500 *μ*m thick wall. We placed the culture insert into a 24-well culture plate and added 1 × 10^4^ cells with or without 50 *μ*M resveratrol treatment into each reservoir for overnight culture. A 500 *μ*m gap was created after removing culture insert. The images of cell migration were captured by a Dino-Lite microscope eye-piece camera (Dino-Lite, Naarden, Netherlands).

### 2.6. Invasion Assay

For invasion assay, we placed Millicell invasion chamber (8 *μ*m pore size, Millipore, Darmstadt, German) with Matrigel (BD Biosciences) into a 24-well plate. In the upper compartment of the invasion chamber, 1 × 10^4^ cells were seeded and filled with 200 *μ*L serum-free DMEM and 50 *μ*M resveratrol. In the lower compartment of the invasion chamber, 600 *μ*L complete DMEM with 10% FBS was added. After 24 hours incubation, the invasive cells located on the underside of the filter were fixed with 3 : 1 ratio of methanol and glacial acetic acid, stained with 2% crystal violet, and counted under a phase-contrast microscope.

### 2.7. Real-Time RT-PCR Analysis

Total RNA was extracted with TRIsure reagent (Bioline Reagents Ltd., London, UK). The concentration and purity of total RNA was determined by NanoDrop ND-1000 Spectrophotometer (NanoDrop Technologies, Inc., Wilmington, DE). Real-time PCR was performed using the SensiFAST SYBR Hi-ROX Kit (Bioline) on the ABI StepOnePlus Real-Time PCR machine (Applied Biosystems, Foster City, CA). The primer sequences used in this study were the same as those described previously [8, 21, 22].

### 2.8. Antibodies for Western Blotting and Immunofluorescence Staining

Antibodies such as anti-*α*-tubulin (1 : 5000, Abcam, Cambridge, MA), anti-actin (1 : 5000, Millipore, Darmstadt, German), anti-Oct3/4 (1 : 1000, Bioworld Technology, Suffolk, UK), anti-E-cadherin (1 : 5000, BD Biosciences), anti-vimentin (1 : 1000, Sigma-Aldrich), anti-p53 (1 : 1000, Millipore), and anti-phospho-p53 (Ser15) (1 : 1000, Cell Signaling, Boston, MA) were used for western blotting and immunofluorescence staining. Western blotting and immunofluorescence staining were performed as described previously [8].

### 2.9. Plasmids, Virus Production, and Infection

Human p53 cDNA was subcloned into pEZ-Lv201-puro lentiviral vector (GeneCopoeia, Rockville, MD, USA). p53 shRNA was cloned in pLKO.1-based lentiviral vector provided by the National RNAi Core Facility, Academia Sinica, Taiwan. We chose the p53 shRNA with the best knockdown efficiency to do further experiment from four p53 shRNAs targeted against different regions of p53. EGFP was cloned into the pAS2-puro-based lentiviral vector provided by the National RNAi Core Facility, Academia Sinica, Taiwan. Utilizing PolyJet DNA *In Vitro* Transfection Reagent (SignaGen Laboratories, Rockville, MD), 293T cells were transfected with viral vectors. The virus-containing supernatant of transfectant was filtered through a 0.45 *μ*m pore size filter 2 days after transfection. Viral infection was achieved by adding virus-containing supernatant supplemented with 8 ng/mL polybrene. Puromycin selection (5 *μ*g/mL) was subsequently employed 24 hours after infection. After one week of puromycin selection, stable clones were used for further analysis.

### 2.10. Measurement of Oxygen Consumption Rate

Oxygen consumption rate was measured by the 782 Oxygen Meter (Strathkelvin Instruments, Motherwell, UK). To measure the steady-state oxygen consumption rate of the cells, 1 × 10^6^ cells with or without 50 *μ*M resveratrol treatment were resuspended in 330 *μ*L of assay buffer (125 mM sucrose, 65 mM KCl, 2 mM MgCl_2_, and 20 mM Na^+^-K^+^-phosphate buffer, pH 7.2) and transferred to a closed chamber of the oxygen meter. To measure the function of the mitochondrial respiratory chain, we first permeabilized the mitochondrial outer membrane by adding 0.002% ethanol-purified digitonin (Sigma-Aldrich) to the chamber (final concentration: 0.0006%). We then injected 6 *μ*L of succinate into the chamber (final concentration: 20 mM). To assay the efficiency of ATP synthesis, we added 1 mM ADP to the chamber to stimulate the respiration. Oxygen consumption rate was also measured with a Seahorse XF24 extracellular Flux analyzer as described by Qian and Van Houten [23]. Cells were seeded in an XF24 cell culture plate and were allowed to grow for one day. The medium was replaced with unbuffered DMEM and incubated in a 37°C incubator for 30 minutes to stabilize the pH and temperature. The oxygen consumption rate (OCR) represented mitochondrial respiration, and the rate of extracellular acidification (ECAR) indicated the lactate production rate by anaerobic glycolysis.

### 2.11. Lactate Production Rate

Cells with or without 50 *μ*M resveratrol treatment were washed with PBS and replenished with fresh medium in a 37°C incubator for 5 hours. An aliquot of 10 *μ*L of medium was then transferred to 96-well dishes and mixed with lactate reagent (Trinity Biotech Plc., Bray, Ireland). The absorbance at 540 nm was measured by an ELISA reader (PowerWave^X^ 340). The lactate production rate was calculated from the absorbance and was normalized by the cell number and incubation time.

### 2.12. Mitochondrial Membrane Potential (Δ*ψ*m)

Cells with or without resveratrol treatment were trypsinized and incubated with 0.25 *μ*g/mL JC-1 fluorescence dye at 37°C in the dark for 15 minutes. The fluorescence intensities of FL1 and FL2 channels were analyzed with Cytomics FC500 flow cytometer (Beckman Coulter, Fullerton, CA).

### 2.13. Intracellular Levels of Reactive Oxygen Species (ROS)

Cells with or without resveratrol treatment were washed by PBS and incubated with a medium containing 40 *μ*M 2′,7′-dichlorofluorescin diacetate (H_2_DCFDA, Invitrogen) at 37°C in the dark for 15 minutes. We then trypsinized the cells and resuspended them in PBS. H_2_DCFDA was used for the determination of intracellular H_2_O_2_. The fluorescence intensity of FL1 channel for H_2_DCFDA staining was analyzed with Cytomics FC500 flow cytometer (Beckman Coulter).

### 2.14. Cell Viability Assay

In the drug treatment experiment, cells were exposed to resveratrol (10, 25, 50, 75, and 100 *μ*M), cisplatin (8 *μ*M, Sigma-Aldrich), or 5-fluorouracil (5-FU; 10 *μ*M, Sigma-Aldrich) for 48 hours. In the irradiation treatment experiment, cells were irradiated (5 Gy or 10 Gy) at 37.9 mGy/s using Rad Source RS 2000 X-ray biological irradiator (Rad Source Technologies, Inc.). After 2 days of culture, cells were washed with PBS, replenished with fresh medium containing 1 × Alamar Blue cell viability assay reagent (AbD Serotec, Oxford, UK), and incubated at 37°C for 2 hours. Fluorescence intensity, with the excitation wavelength at 538 nm and the emission wavelength at 590 nm, was measured using a Fluoroskan Ascent Microplate Fluorometer (Thermo Fisher Scientific, Inc., Waltham, MA).

### 2.15. *In Vivo* Analysis of the Tumor Curability by Resveratrol

To monitor the *in vivo* growth of CSCs, we first established the GFP-CSCs by transfecting a pAS2-EGFP-puro expression construct to NPC CSCs. A total of 100 GFP-CSCs were then injected into hypoglossal regions of 7-8-week-old NOD/SCID mice. In the group of resveratrol treatment, we fed mice with 100 mg/kg resveratrol every 2 days after injecting the CSCs. The incidence of tumor growth was measured after 2 weeks. The mice were sacrificed, and the distribution of GFP-positive tumors was observed through Youlum Sky-blue II epifluorescent light (Youlum Inc., New Taipei City, Taiwan).

### 2.16. Statistical Analysis

All data are presented as mean ± SD. Statistical analysis was performed by using Student's *t*-test and a difference between groups with *P* < 0.05 is considered significant.

## 3. Results

### 3.1. NPC CSCs Exhibit Characteristics of Stemness, EMT, and Metabolic Reprogramming

**Figure 1:**
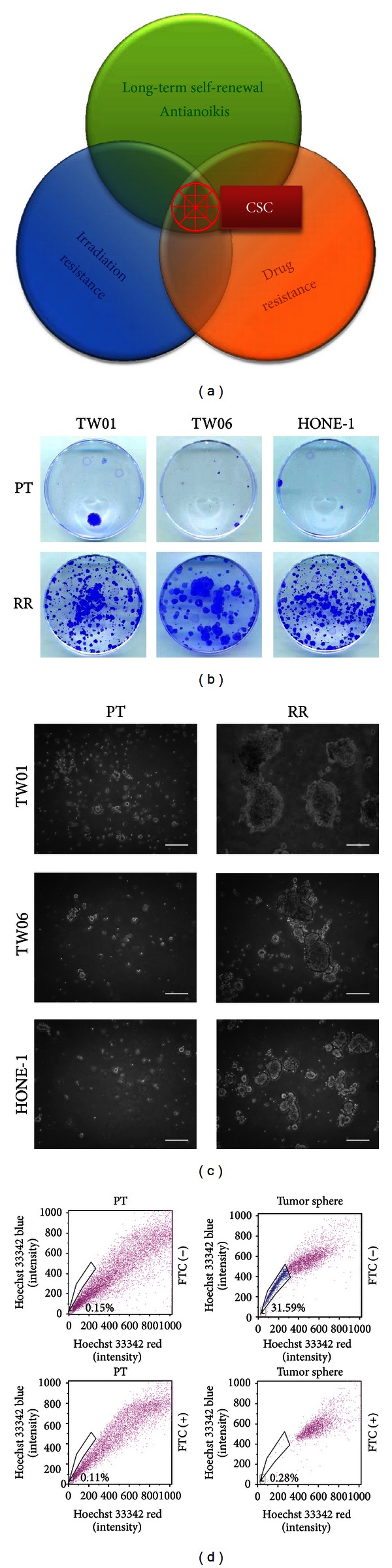
The concept and process validation of NPC CSC isolation achieved by behavior selection. (a) The concept of behavior selection is based on CSC properties, which are resistance to irradiation and drugs, long-term self-renewal, and antianoikis. Through the behavior selection platform, abundant and high-purity CSCs could be obtained. (b) After four rounds of 11 Gy irradiation selection, radioresistant clones (RR) of TW01, TW06, and HONE-1 were established. Further colony formation assays of these radioresistant cells and parental cells (PT) were performed with seeding 5 × 10^3^ cells after 10 Gy irradiation. Representative images are macroscopically visible survival colonies after fixation and crystal violet staining. Radioresistant cells show distinct radioresistant phenotype. (c) After irradiation selection, tumor sphere selection was performed. Compared with parental cells, radioresistant clones could generate more and larger spheres. Scale bars indicate 100 *μ*m. (d) Side population assay shows that TW01 parental cells (left panel) contained less side population cells compared with tumor sphere cells (right panel). The top panel represents the cells incubated with Hoechst 33342. A subset of side population cells pumped out of the Hoechst dye by ABC transporters and demonstrated low fluorescence expression. The bottom panel shows that the gating proportion is reduced from 0.15% to 0.11% in parental cells and from 31.59% to 0.28% in tumor sphere cells after fumitremorgin C (FTC) treatment. FTC can block the ABC transporters and result in full side population efflux inhibition.

**Figure 2:**
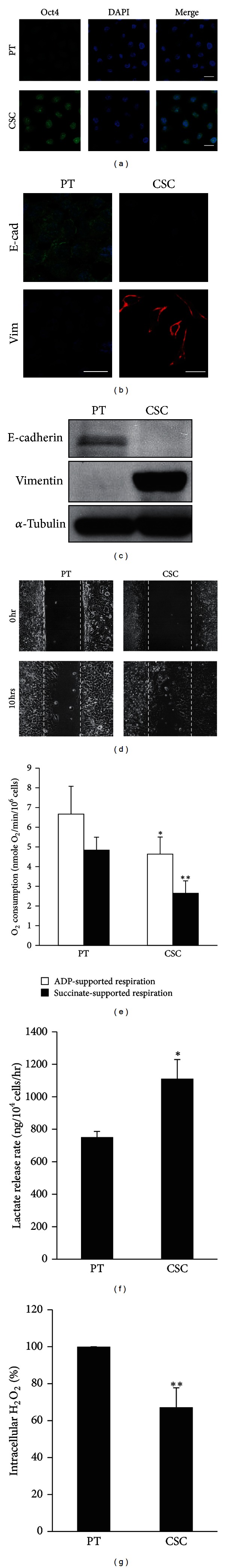
NPC CSCs manifested characteristics of stemness, EMT, and metabolic reprogramming. (a) Images of immunofluorescence staining for Oct4 expression show overexpression and nuclear translocation of Oct4 in TW01 CSCs compared with the negative control of TW01 parental cells. (b) Immunofluorescence staining indicates the transition of epithelial marker (E-cad: E-cadherin) and mesenchymal marker (Vim: Vimentin) in TW01 parental cells and CSCs. Scale bars in (a) and (b) indicate 25 *μ*m. (c) Western blots confirm the EMT of TW01 CSCs at the protein level. (d) Wound healing assay showed higher migratory behavior of TW01 CSCs compared with TW01 parental cells. Full wound closure was observed in TW01 CSCs after 10 hours. (e) The O_2_ consumption rates supported by ADP and succinate were lower in TW01 CSCs compared with TW01 parental cells. (f) TW01 CSCs had higher levels of lactate production than did TW01 parental cells. (g) Intracellular H_2_O_2_ level was relatively lower in TW01 CSCs compared with that in TW01 parental cells measured by H_2_DCFDA staining. (*P < 0.05; **P < 0.01).

The most common methods used to isolate CSCs focus on single CSC ability, such as side population or tumor sphere. To improve upon these current methods, we combined irradiation selection, tumor sphere selection, and side population selection to isolate CSCs. The three methods overlapped to form a bull's eye area where purity of CSCs may be the highest (Figure 1(a)). We started with establishing NPC radioresistant clones (Figure 1(b)) by four rounds of high-dose irradiation selection. Several radioresistant NPC clones were established. These radioresistant clones underwent tumor sphere selection and displayed higher tumor sphere formation capacity compared with parental cells (Figure 1(c)). The percentage of side population is greatly higher in tumor sphere cells than that in parental cells (Figure 1(d)). It signified that irradiation selection can increase the feasibility to obtain CSC-like cells as compared with the feasibility of isolating CSC-like cells directly from parental cells merely by tumor sphere or side population selection. Following behavior selection, the CSC clones became resistant to radiotherapy and chemotherapy and capable of forming tumor spheres. Stemness-related transcription factor Oct4 was expressed and translocated from cytoplasm to the nucleus of isolated CSCs (Figure 2(a)). This implied that nuclear Oct4 might activate its target genes and result in stem cell-like features of CSCs. CSCs also underwent an EMT with downregulation of epithelial marker E-cadherin and concomitant upregulation of mesenchymal marker vimentin (Figures 2(b) and 2(c)). CSCs manifested greater migratory capacity than did parental NPC cells (Figure 2(d)). To know the metabolic characteristics, we then investigated the organelle function of NPC CSCs. The O_2_ consumption rates driven by ADP and succinate were lower in CSCs compared with those in parental NPC cells (Figure 2(e)), which implied that these CSCs might not rely on mitochondrial respiration to produce energy. Likewise, CSCs excreted higher levels of lactic acid as a by-product than did parental NPC cells (Figure 2(f)). Moreover, the level of prooxidants was relatively lower in CSCs compared with parental NPC cells through analysis of intracellular H_2_O_2_ with H_2_DCFH-DA (Figure 2(g)). These findings revealed that CSCs displayed unique metabolic signatures similar to those of stem cells.

### 3.2. Resveratrol Suppresses the Stemness, EMT, and Metabolic Reprogramming in CSCs

**Figure 3:**
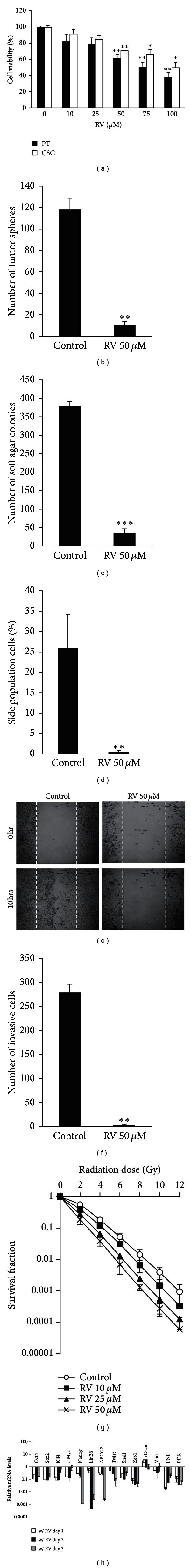
Resveratrol suppressed CSC properties. (a) Evaluation of cytotoxic effects of different concentration of resveratrol (RV) in TW01 parental cells and CSCs. Significant effects are shown with concentration more than 50 *μ*M. Capacities for (b) tumor sphere formation, (c) soft agar colony formation, and (d) percentage of the side population were suppressed in TW01 CSCs after 50 *μ*M resveratrol treatment. (e) Migration capacity analyzed by wound healing assay and (f) invasive capacity measured by transwell invasion assay were suppressed by 50 *μ*M resveratrol in TW01 CSCs. (g) Resveratrol could increase the radiosensitivity of TW01 CSCs in a concentration-dependent manner measured by survival fraction assay. (h) Resveratrol (50 *μ*M) altered the expression of indicated genes in TW01 CSCs. E-cad: E-cadherin; N-cad: N-cadherin; Vim: vimentin; FN1: fibronectin 1; PDK: pyruvate dehydrogenase kinase. Data were normalized with the mRNA expression level of 18*S* rRNA and compared with those of TW01 CSCs without resveratrol treatment on a log scale. (*P < 0.05; **P < 0.01; ***P < 0.001).

**Figure 4:**
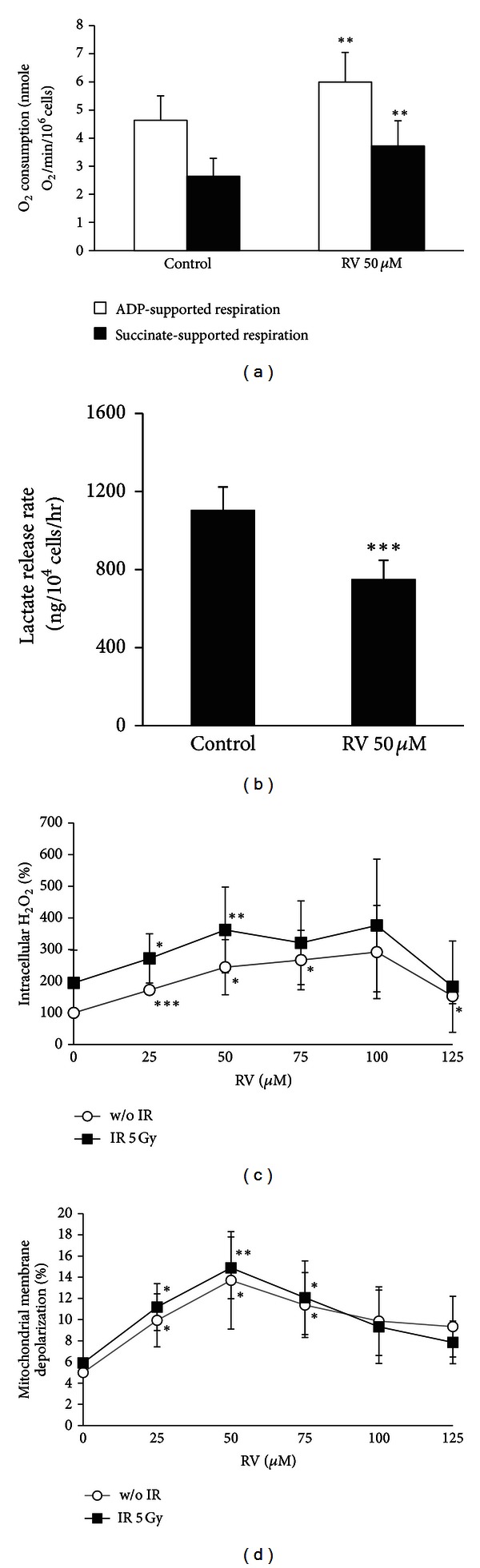
Resveratrol could reverse the metabolic reprogramming of CSCs. (a) Resveratrol (50 *μ*M) increased O_2_ consumption and (b) repressed the lactate production of TW01 CSCs. (c) Intracellular H_2_O_2_ was upregulated in TW01 CSCs exposed to serial doses of resveratrol and 5 Gy irradiation (IR) measured by H_2_DCFDA staining. (d) Serial doses of resveratrol depolarized the mitochondrial membrane in TW01 CSCs with or without 5 Gy irradiation. The percentage of mitochondrial membrane potential depolarization was measured by the JC-1 green fluorescence shift. In (c) and (d) charts, TW01 CSCs with resveratrol treatment were compared with those of TW01 CSCs without resveratrol treatment. TW01 CSCs with both resveratrol and irradiation treatment were compared with those of TW01 CSCs with irradiation but without resveratrol treatment. (*P < 0.05; **P < 0.01; ***P < 0.001).

The versatile efficacy of resveratrol on NPC CSCs was unraveled in this study. We first evaluated the sensitivity of parental cells or CSCs under different concentrations of resveratrol treatment. Cell viability was significantly affected if the concentration of resveratrol was higher than 50 *μ*M in parental cells or CSCs (Figure 3(a)). We then mainly used 50 *μ*M resveratrol in following assays. Resveratrol could suppress the long-term self-renewal and antianoikis capacity (Figure 3(b)) and *in vitro* tumorigenicity (Figure 3(c)). Resveratrol also significantly reduced the percentage of side population (Figure 3(d)) and migration and invasion capacity in CSCs (Figures 3(e) and 3(f)). Combining irradiation with resveratrol increased the radiosensitivity of CSCs which suggested that resveratrol could potentially serve as an irradiation sensitizer (Figure 3(g)). In addition, resveratrol diminished the expression of genes characteristic of stemness, EMT, and metabolism (Figure 3(h)). Downregulation of stemness-related genes such as Oct4, Sox2, Klf4, c-Myc, Nanog, and Lin28 depicted that resveratrol compelled the differentiation of CSCs. Under resveratrol treatment, ABCG2 suppression also increased the drug sensitivity of CSCs. In addition, resveratrol reversed the EMT phenotype of CSCs by inhibiting the expression of EMT regulators and mesenchymal markers, such as Twist, Snail, Zeb1, vimentin, and fibronectin; on the contrary, epithelial marker E-cadherin was increased. Moreover, downregulation of pyruvate dehydrogenase kinase (PDK), a negative regulator of pyruvate dehydrogenase (PDH), promoted pyruvate back to mitochondrial biosynthetic pathways in CSCs. As expected, increased O_2_ consumption and decreased lactate production demonstrated that resveratrol reversed the metabolic shift in NPC CSCs (Figures 4(a) and 4(b)). Resveratrol also significantly increased the ROS level and depolarized mitochondrial membranes in CSCs especially under irradiation treatment (Figures 4(c) and 4(d)).

### 3.3. Resveratrol Impedes CSC Properties through p53 Activation

**Figure 5:**
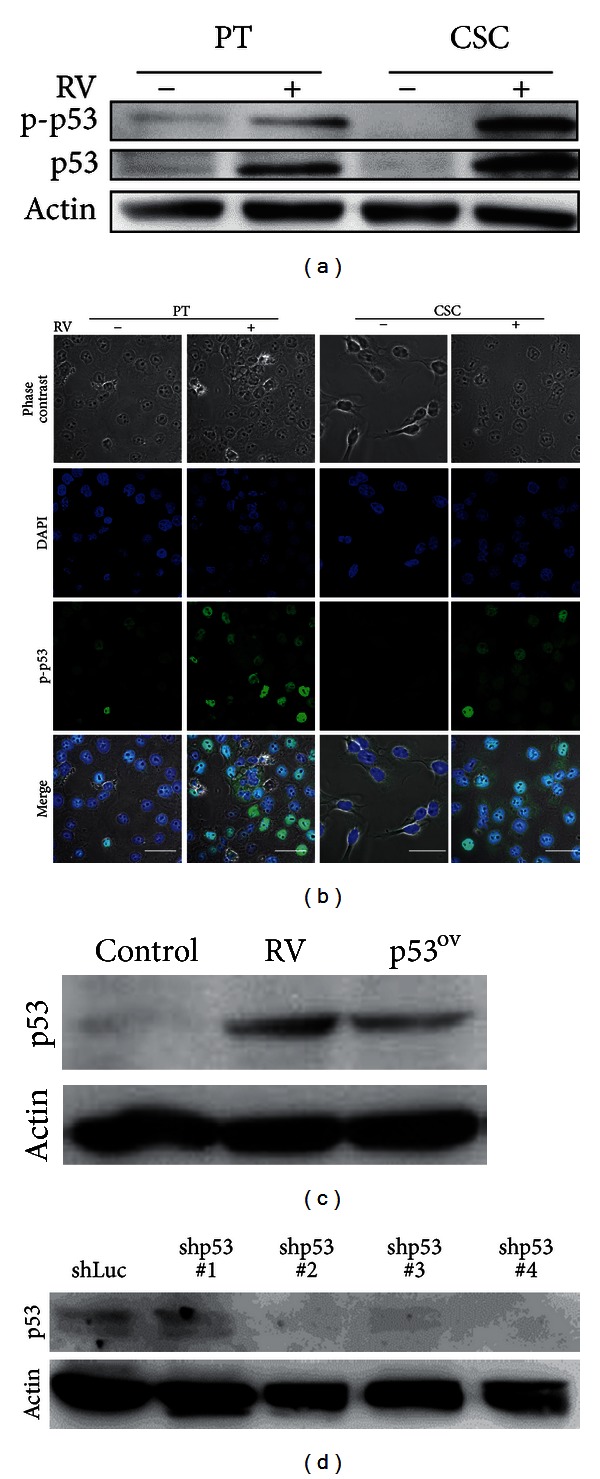
Resveratrol impeded EMT through p53 activation in CSCs. (a) Expressions of p53 and phosphorylated p53 (Ser15) were lower in TW01 CSC than those in TW01 parental cells. Resveratrol could increase expression of p53 and phosphorylated p53 expression both in CSC and parental cells. (b) Resveratrol (50 *μ*M) reactivated p53, indicated by the translocation of phosphorylated p53 (Ser15) from cytoplasm to the nucleus (detected with DAPI staining), and repressed the EMT phenotype in TW01 CSCs. TW01 parental cells remained epithelial type after resveratrol treatment. Scale bars indicate 40 *μ*m. (c) Western blot indicated the p53 expression of TW01 CSCs with 50 *μ*M resveratrol treatment (RV) or p53 overexpression (p53^ov^). (d) Western blot confirmed the p53 knockdown efficiency in TW01 CSCs. shp53#2 with the best knockdown efficiency was chose for further experiment.

**Figure 6:**
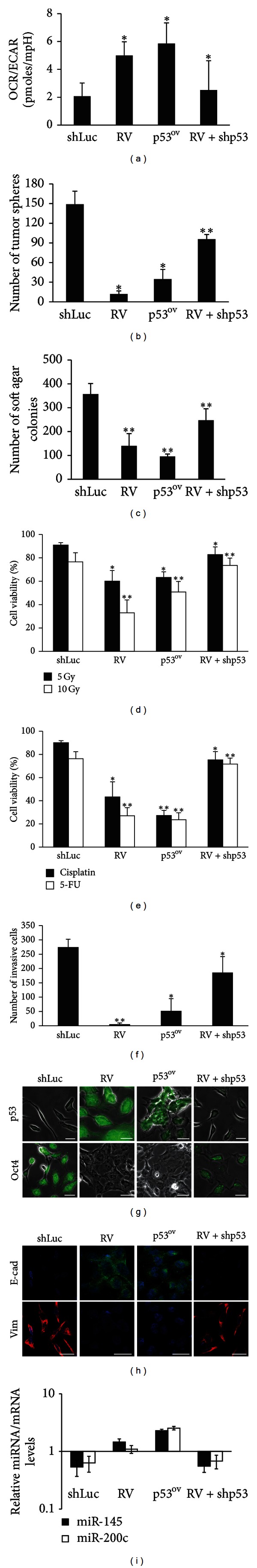
Loss of p53 expression reversed the effects of resveratrol on CSCs. Resveratrol and p53 overexpression had similar effects on TW01 CSCs in (a) OCR/ECAR ratio measurement, (b) tumor sphere formation assay, (c) soft agar assay, (d) cell viability assay under 5 Gy and 10 Gy irradiation, (e) cell viability assay under cisplatin (8 *μ*M) and 5-fluorouracil (5-FU; 10 *μ*M) treatment, and (f) invasive capacity assay. Repressing p53 expression diminished resveratrol effects. (g) Resveratrol and p53 overexpression suppressed Oct4 expression in TW01 CSCs. Loss of p53 expression reversed the effects of resveratrol. (h) Similarly, resveratrol and p53 overexpression reversed EMT process with increased E-cadherin (E-cad) and decreased vimentin (Vim) expression. Loss of p53 expression reversed the effects of resveratrol. Scale bars in (g) and (h) indicate 25 *μ*m. (i) Upregulation of miR-145 and miR-200c was detected in resveratrol-treated and p53 overexpressed CSCs. Blocking p53 expression could reverse their expressions. Results were normalized with *RNU6B* and compared with that in TW01 parental cells. (shLuc: CSCs with shLuc as a control; RV: CSCs with resveratrol treatment; p53^ov^: CSCs with p53 overexpression; RV + shp53: resveratrol-treated CSCs with p53 knockdown; RV group and p53^ov^ group were compared with shLuc group; RV + shp53 group was compared with RV group; *P < 0.05; **P < 0.01).

We found that p53 expression level was lower in CSCs than that in parental cells (Figure 5(a)). Furthermore, the mesenchymal cell shape of CSCs was reversed to epithelial type concomitant with the activation of p53 (phosphorylated at serine 15) after resveratrol treatment (Figures 5(a) and 5(b)). To confirm the idea that resveratrol could suppress the CSC properties directly through the induction of p53, we modulated the expression of p53 in CSCs by infecting a lentivirus-based full-length p53 clone and shRNAs targeting p53, respectively (Figures 5(c) and 5(d)). We then measured the oxygen consumption rate (OCR) and extracellular acid efflux rate (ECAR) of these CSCs by a Seahorse XF24 Extracellular Flux Analyzer. OCR is an indicator of mitochondrial respiration, and ECAR is predominantly a measure of lactic acid production rate during glycolysis. Similar to the effects of resveratrol, overexpression of p53 increased the OCR/ECAR ratio in CSCs; knockdown of p53 reduced the OCR/ECAR ratio during resveratrol treatment (Figure 6(a)). These data substantiated the notion that resveratrol altered the energy metabolism mainly by induction of p53. Remarkably, suppression of tumor sphere formation and soft agar colony formation achieved by resveratrol treatment could be rescued by knocking down p53 (Figures 6(b) and 6(c)). Moreover, the sensitivity to irradiation and chemotherapeutic agents induced by resveratrol could be blocked by knocking down p53 (Figures 6(d) and 6(e)). Invasive capacity was also found to be derepressed after p53 knockdown in resveratrol-treated CSCs (Figure 6(f)). As anticipated, resveratrol and p53 overexpression reduced the expression of stemness and EMT, while the silencing of p53 restored the expression of stemness and EMT in resveratrol-treated CSCs (Figures 6(g) and 6(h)). In addition, we noticed that miR-145 and miR-200c were downregulated in CSCs but could be upregulated by resveratrol treatment and p53 overexpression (Figure 6(i)). The upregulation of miR-145 and miR-200c could be attenuated by knockdown of p53 in resveratrol-treated CSCs (Figure 6(i)).

### 3.4. Resveratrol Annihilates CSCs in NOD/SCID Mice

**Figure 7:**
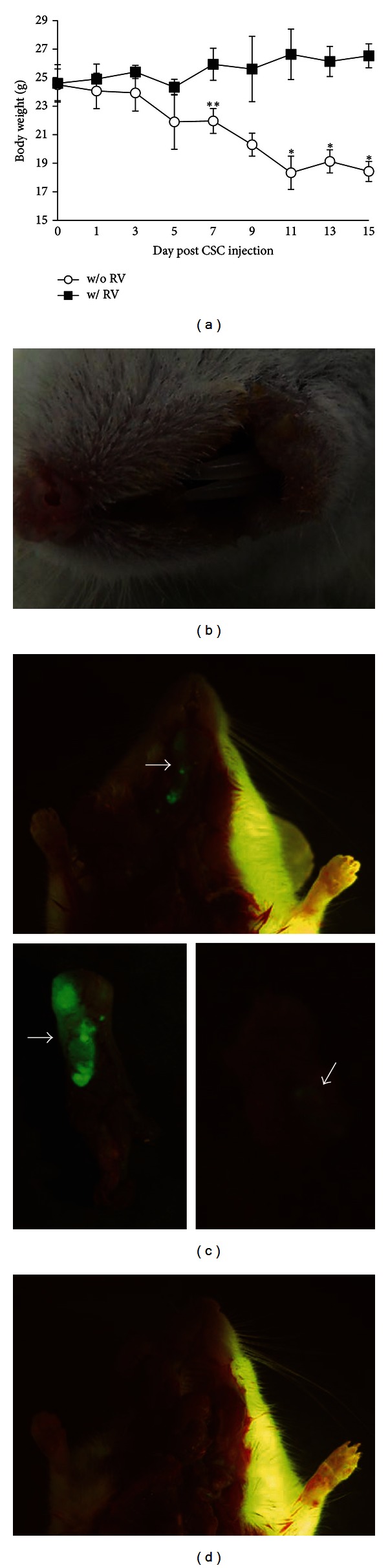
Resveratrol suppressed the *in vivo* growth of CSCs. (a) Mice without resveratrol feeding (w/o RV) were 6 g thinner in body weight as compared with the mice with resveratrol feeding (w/ RV) after 2 weeks. (b) By hypoglossal injection and without resveratrol feeding, the indurate tumor lumps disseminated around NOD/SCID mouse's buccal mucosa after two weeks. (c) By hypoglossal injection and without resveratrol feeding for two weeks, GFP-lentivirus-infected TW01 CSCs grew throughout the tongue (bottom left) and lower jaw (bottom right) of the mouse, as seen under a Sky-blue II epifluorescent light (tumor size 50–270 mm^3^). (d) As seen under a Sky-blue II epifluorescent light, TW01 CSCs were eradicated in the hypoglossal region of the mouse tube-fed with resveratrol after two weeks. (**P < 0.05; **P < 0.01).

Lastly, we examined the curative effect of resveratrol *in vivo*. We injected GFP-CSCs into the hypoglossal region of NOD/SCID mice. The mouse without resveratrol feeding was asthenic in appearance, lost their body weight gradually, and tumor lumps disseminated around the bottom of buccal mucosa (Figures 7(a) and 7(b)). By *in vivo *GFP tracking, we observed that tumor grew throughout the tongue and lower jaw of the mice without resveratrol treatment (Figure 7(c)). The CSCs were finally eradicated, and the mice stayed healthy when the mice fed with a diet supplemented with resveratrol (Figure 7(d)).

## 4. Discussion

Metabolism has been the focus of cancer research as early as the 1920s, when Otto Warburg observed that malignant tissues produce energy predominately by glycolysis rather than oxidative phosphorylation (OXPHOS) even when oxygen is plentiful [24]. This metabolic shift from aerobic metabolism to glycolysis also occurs in embryonic stem cells (ESCs) and induced pluripotent stem cells (iPSCs) [25]. Similar to normal stem cells, breast CSCs contain a lower level of ROS compared with nontumorigenic cancer cells [26]. To date, the metabolic signatures of CSCs remain largely unknown. Our findings revealed that CSCs, like normal stem cells, underwent metabolic shift from aerobic metabolism to anaerobic glycolysis (Figures 2(e) and 2(f)). Moreover, the levels of ROS were lower in NPC CSCs that may result in radioresistance as previously reported in breast CSCs (Figures 1(b) and 2(g)).

Abundant evidence has been accumulated to substantiate the anticancer activities of resveratrol in various human cancers [27–30]. We found that resveratrol could turn off the metabolic switch, increased the ROS level, and depolarized mitochondrial membranes in NPC CSCs (Figure 4). These alterations in metabolism occurred concomitantly with the suppression of the CSC properties including the resistance to radiotherapy and chemotherapy, self-renewal capacity, tumor initiation capacity, and metastatic potential in NPC CSCs (Figure 3). Particularly worth mentioning is that resveratrol tackled the nexus of NPC CSCs which resulted in extensive suppression of stemness, EMT, and metabolism-related genes (Figure 3(h)). This extensive suppression in CSCs could also be observed after we had ectopically expressed p53, the downstream target of resveratrol (Figure 6). In addition, the suppression of CSC properties by resveratrol could be attenuated by knocking down p53 (Figure 6). These findings substantiated the notion that p53 may serve as a common link between metabolism, stemness, and EMT in CSCs.

It was reported that resveratrol can increase the p53 protein level in breast cancer cell line without altering the p53 mRNA levels, suggesting that resveratrol may still be useful to treat tumors with a loss of normal p53 function [31]. Besides, resveratrol could significantly activate intracellular Notch-1 and restore wild-type p53 expression in glioblastoma cells [32]. These findings indicate that resveratrol may be an effective drug for treatment of tumors without normal p53 function.

Recent studies have revealed a connection between p53 and stem cell biology, which signified the importance of p53 pathway in CSCs [33, 34]. Several studies indicated that p53 pathway decreases the efficiency of reprogramming of somatic cells to iPSCs [35–39]. The absence of p53 allows the suboptimal cells to become iPSCs and also accelerates nuclear reprogramming by loss of p53-dependent cell cycle arrest. The iPSCs with functional p53 were able to form teratoma which differentiated into three germ layers. However, the absence of p53 seemed to be the tradeoff between reprogramming efficiency and tumorigenesis. The teratoma of iPSCs with p53 knockout or a mutation in p53 contained undifferentiated tissue cells which resembled tumor growth [35]. Besides, p53 could control the MDM2-mediated slug degradation [40] and suppress c-Myc through the induction of miR-145 [41]. p53 also could inhibit the expression of CD44 by modulating miR-34a in prostate CSCs [42]. Moreover, p53 was able to launch miR-200c to suppress genes that mediate EMT and stemness properties [43].

These findings suggest that p53 has a significant role in connecting reprogramming to stemness status and tumorigenesis of CSCs. In ESC studies, researchers found that miR-145 suppressed the expression of Oct4, Sox2, and Klf4 [44]. In cancer studies, researchers found that miR-145 could be considered as a tumor suppressor and was downregulated in several cancers including NPC [45]. Since resveratrol-induced p53 is able to transcriptionally activate the expression of miR-145 and miR-200c (Figure 6(i)), the role of p53 in suppressing the stemness and EMT via induction of miRNAs awaits further investigation. Through the elucidation of the p53/miRNAs mechanism, we may be able to apply this regulation in the therapy targeting at CSCs.

## 5. Conclusions

Thus far, the link between metabolic reprogramming, stemness, and EMT of CSCs has remained elusive. However, this link appears to be of vital importance as uncovered by the aforementioned effects of resveratrol. The regulation of p53 within stemness, EMT, and metabolic reprogramming should be further investigated with the hope that it may lead to the identification of novel therapeutic targets for future anticancer therapies in humans.
